# Editorial: Total marrow irradiation

**DOI:** 10.3389/fonc.2023.1240530

**Published:** 2023-07-19

**Authors:** Susanta Kumar Hui, Guy Storme, Jeffrey Wong, Cynthia Aristei, Monzr M. Al Malki, Bulent Aydogan

**Affiliations:** ^1^ Department of Radiation Oncology, Beckman Research Center, City of Hope National Medical Center, Duarte, CA, United States; ^2^ Department of Radiation Oncology, Vrije University Brussels, Brussels, Belgium; ^3^ Department of Radiation Oncology, City of Hope National Medical Center, Duarte, CA, United States; ^4^ Department of Radiation Oncology, University of Perugia, Perugia, Perugia, Umbria, Italy; ^5^ Department of Hematology and Hematopoietic Cell Transplantation, City of Hope National Medical Center, Duarte, CA, United States; ^6^ Department of Radiation and Cellular Oncology, University of Chicago, Pritzker School of Medicine, Chicago, IL, United States

**Keywords:** TMI, TBI, TMLI, conformal TBI, IMRT TBI

Although total body irradiation (TBI) is commonly used in conditioning regimens for allogeneic hematopoietic cell transplantation (HCT) in hematological diseases. major drawbacks are treatment-related toxicities and relapse. Despite advances in precision radiation for solid tumors, TBI for HCT has remained practically unchanged for over half a century due to a lack of i) advances in treatment delivery, 3D treatment planning, and organ-specific dosimetry, ii) limited understanding of the biological impact of systemic and targeted radiation, iii) appropriate clinical studies addressing innovative issues in hematological diseases and iv) integration across multidisciplinary fields to solve clinical uncertainties. The field of TBI has begun shifting from conventional TBI strategies towards 3D image-guided organ-specific treatment delivery i.e. “total marrow irradiation (TMI)”, “total marrow and lymphoid radiation (TMLI)”, “conformal whole-body irradiation” or intensity-modulated TMI (IMTMI). Despite subtle differences, all these treatments are referred to as TMI. Acceptance of TMI regimens is accelerating worldwide as TMI equipment is available from diverse manufacturers.

Since a full report about progress in this multidisciplinary field is acknowledged to be an unmet need, despite several individual studies, the present special issue was designed to cover recent advances in TMI technology, physics, biology, imaging, and clinical benefits. The series also reports on TMI standardization across manufacturers through a data-collecting consortium. Additionally, future advances through a multidisciplinary approach, including molecular imaging and automatization, will be achieved by bringing together international experts in different disciplines. Therefore, this project aims to be transformational, as it will cover advances in all aspects of this new field, inform about scientific progress, and guide clinical practice.

This Research Topic contains 15 articles covering a wide range of topics that were written by 238 contributors. They mainly fall into 4 categories:

1) Clinical studies
○ Vogel et al. reviewed the most common adverse effect of TBI i.e. pulmonary toxicity including the idiopathic pneumonia syndrome (IPS), emphasizing that definitions of IPS as well as demographic and treatment-related risk factors remain poorly characterized. Indeed, few data correlated dose distribution with toxicity. In the future, CT-guided intensity modulated TBI is expected to provide extremely precise calculations of 3D lung dose distributions in order to correlate dose volume histograms with toxicity. The authors suggested assessing risk factors for IPS in cohorts of pediatric and adult patients and adopting the diagnostic workup and definition as proposed by the American Thoracic Society.
○ Wong et al. analyzed data from over 500 patients who received, as part of conditioning regimens to various HCT, TMLI delivered by Tomotherapy or Linac-based VMAT. In a Phase II study, TMLI dose escalation to 20 Gy, combined with etoposide and cyclophosphamide, improved outcomes (2-year OS and PFS 48% and 33%) with 1-year non-relapse mortality (NRM) of 6%). Another innovation was the breakthrough development of chemotherapy-free conditioning based on 20 Gy TMLI followed by PTCy. It was designed to reduce the risk of GVHD while maintaining a high antileukemic effect in patients with AML in CR1/CR2 undergoing matched donor allogeneic HCT. Promising 2 year results were reported: OS =86.7%, relapse-free survival = 83.3%, GVHD-/relapse-free survival (GRFS) = 59.3% and no NRM. In conclusion, TMI/TMLI allowed dose escalation, was suitable for elderly patients, and was associated with good outcomes.
2) Clinical trials
○ Kobyzeva et al. treated a large cohort of children with leukemia with conformal TBI (12 Gy delivered in 6 or 4 fractions) The dose to the bone marrow was increased up to 15 Gy in a small cohort. All patients received standard chemotherapy followed by TCR-alpha/beta-depleted donor marrow to minimize the risk of GVHD and 88% received a haploidentical transplant. At a median follow-up of over 2 years, OS was 63%, and TRM 10.7%. Disease status heterogeneity could potentially have impacted outcomes. In patients with active disease increasing the BM-targeted radiation dose reduced disease recurrences and improved survival (47% vs 29%), suggesting higher BM-targeted radiation doses are needed to control enhanced disease burden in the bone marrow.
○ Ladbury et al. retrospectively evaluated the effects of RT on outcomes in 254 patients with refractory or relapsed AML or ALL who suffered extramedullary (EM) relapse after TMLI. RT was delivered with curative intent to 11 patients in whom significantly better OS and PFS were observed. The authors concluded RT effectively treated EM relapse, particularly if limited.
○ Kong et al. analyzed the feasibility and effectiveness of 12 Gy TMLI in the conditioning regimen for allogeneic HSCT in a small series of 1 patient with AML and 16 with ALL (median age 17 years; range 8-35). Although this pilot study showed TMLI was safe, a longer follow-up is needed to assess outcomes.
○ Saldi et al. retrospectively analyzed the main dosimetric parameters to determine the impact of RT doses to the intestine on the incidence of acute GvHD (aGvHD) in transplant recipients. No dosimetric parameter was associated with aGvHD, not even when the intestine was divided into sub-areas. The limitations of this study were a large dose variation in the intestine and a small cohort of patients. Furthermore, transplants and patient ages were mixed (HLA matched and HLA haploidentical transplants; young adults and older patients). As all patients received adoptive immunotherapy with Tcons and Tregs, untangling the role of RT was complex. Thus, a preclinical model was needed to elucidate the role of TMI in aGvHD occurrence. Indeed, lowering the radiation dose (~4 Gy) to the GI attenuated tissue damage, with less donor T-cell traffic to the GI system, resulting in reduced aGvHD [Sargur Madabushi et al. (2022), 140(Supplement 1):4467-4469].
3) Physics and dosimetry
○ Using phantom and simulated motion, Kavak et al. studied the impact of respiratory motion on the lung dose, finding that it may impact small lung regions, but has a negligible effect on dose uncertainty.
○ Loginova et al. provided treatment planning details for TBI, comparing results in children treated with Tomotherapy (157) or VMAT (52); image-guided RT was used in all cases. Compared with VMAT, Tomotherapy displayed less variation between planned and delivered doses, was less time-consuming, and was easier to implement. Since both techniques were feasible, safe, and associated with acceptable toxicity rates, treatment can be performed with either.
○ Ladbury et al. reported treatment planning and dosimetry with VMAT, showing they were similar to previous observations with Tomotherapy and confirming VMAT is suitable for TMI treatment, even at doses up to 20Gy.
○ Han et al. evaluated dosimetric coverage for targets and organs at risk when TMLI was delivered with the standard 12 Gy or 20 Gy. Mean and median doses for most normal organs at the escalated prescription dose of 20 Gy were increased less than the prescription dose scaling.
○ Since TMI/TMLI requires extensive contouring of target volumes and organs of the entire body. As the contouring procedure is time-consuming and prone to errors, it constitutes a major barrier to clinical implementation, Watkins et al. developed a model of Artificial-Intelligence segmentation which offered a powerful solution for enhanced efficacy in TMLI treatment planning.
○ Zuro et al. compared the dosimetric results of TMI treatment planning with intensity-modulated spot-scanning proton therapy (IMPT) and VMAT using photon beams. Except for the esophagus and thyroid, OAR doses were lower with IMPT, and higher for the skull surface and ribs. Nowadays, since IMPT is used for craniospinal irradiation (CSI) in hematological malignancies, a shift to TMI may be feasible in the near future, particularly for pediatric patients.
In summary, TMI treatment is delivered by means of machines from leading manufacturers, thus facilitating clinical studies worldwide.
4) Scientific advances
○ Conformal radiation delivery in a preclinical model is extremely challenging as some vital organs are very close to the skeleton. Moreover, treatment delivery is long and complex. Abdelhamid et al. showed a novel Sparse Orthogonal Collimator (SOC) based intensity modulation for TMI treatment planning and delivery optimization which could enhance dosimetric conformality, reduce radiation exposures to all critical organs, automatize and shorten treatment delivery time.
○ While the benefits of TMI are beginning to emerge in young adults and children, its role in treating older patients remains unknown. Myeloablative radiation has not been used in the elderly due to concerns that increased radiation may adversely affect bone marrow hematopoiesis and that its toxicity profile is unknown. Using preclinical TMI-based dose escalation in aging mice models, Lim et al. observed normal donor engraftment, significantly reduced tissue damage and preserved repair capacity.
○ HCT offers a curative option for Sickle Cell Disease (SCD), a serious global health problem. Myeloablative TBI-based HCT is, however, associated with high mortality/morbidity rates. Conversely, RIC is associated with fewer organ toxicities, but a higher risk of graft rejection. Although it provides mixed chimerism, the donor component gradually reduces over time, leading to SCD relapse. Using a preclinical TMI-based SCD mice model, Madabushi et al. observed that increased BM-targeted radiation enhanced chimerism and stable engraftment, rescued red blood cells from sickle abnormalities, and significantly reduced organ toxicity.
In summary, preclinical models justify initiating new clinical trials for older patients (>55) with leukemia (NCT03494569) and patients with severe sickle cell disease (NCT05384756), such as those currently underway at the City of Hope.

## Author contributions

All authors listed have made a substantial, direct, and intellectual contribution to the work and approved it for publication.
